# Exploring Advance Care Planning in Taiwanese Indigenous Cancer Survivors: Proposal for a Pilot Case-Control Study

**DOI:** 10.2196/resprot.5428

**Published:** 2017-12-21

**Authors:** In-Fun Li, Yvonne Hsiung

**Affiliations:** ^1^ Mackay Memorial Hospital New Taipei City Taiwan; ^2^ Department of Nursing Mackay Medical College New Taipei City Taiwan

**Keywords:** Taiwanese indigenous peoples, advance care planning, cancer survivors

## Abstract

**Background:**

Research on Taiwanese indigenous cancer survivors’ end-of-life (EOL) planning is still in its infancy, despite recent government and societal efforts to promote quality EOL care. Previous national studies in Taiwan have characterized indigenous peoples as a socioeconomically disadvantaged minority group. Compared with their mainstream cohorts, these remote residents are vulnerable to multiple social welfare problems, receiving and accessing little in the way of health care in rural mountain areas. Although advance care planning (ACP) has been shown to help patients achieve better quality of dying, very little is known about indigenous intentions for such interventions. Relevant studies are scarce in Taiwan, and programs for cancer survivors have been based almost entirely on nonindigenous populations. Since there has been no research on Taiwanese indigenous people’s aims for ACP, there is a need to understand the impact of survivorship on ACP readiness among those who are currently living with, through, and beyond cancer.

**Objective:**

We aim to identify differences in ACP intent and readiness among indigenous peoples with and without cancer diagnoses. We will identify the impact of factors such as tribal cultural beliefs and quality of life along with cancer exposure on the outcome of ACP readiness differences. In particular, we will examine the effects of ACP knowledge from previous ACP participation, EOL care experiences, and personal registry status of Do-Not-Resuscitate (DNR) in the national database. A secondary objective is to describe indigenous people’s intent to participate in public education related to EOL planning.

**Methods:**

A descriptive case-control study (N=200) is proposed where controls are matched to cases’ attributes of age, gender, and cancer diagnosis. This matching analysis allows assessment of cancer as an exposure while taking into account age and gender as confounding variables. We are currently in the process of training personnel and extracting clinical and administrative information from the health care system of collaborating facilities. This carefully designed study provides a unique opportunity because for the first time in Taiwan, cancer survivorship and ACP readiness for EOL planning will be examined among difficult-to-reach indigenous peoples.

**Results:**

We plan to complete this study in approximately 3 years.

**Conclusions:**

In this study, we expect to survey palliative care usage in the remote indigenous group, understand factors that influence ACP readiness, and later foster culturally appropriate ACP public participation and policies in order to facilitate collaboration between cancer health care providers in various Taiwanese subcultures.

## Introduction

Evidence has shown that successful interventions promoting advance care planning (ACP) have helped patients achieve their end-of-life (EOL) care goals [1], increase satisfaction [2], reduce chances of over- or undertreatment at EOL [3], and minimize conflicts between family members and health care providers during EOL discussions [4].

Despite the national emphasis on minority health and palliative care in Taiwan [5], research on terminal indigenous patients’ EOL treatment and care options is still premature [6]. Our understanding of their ACP knowledge and intentions remain unclear, as well as their cultural attitudinal preferences and behavioral engagement [6]. In the most recent Taiwanese Indigenous Health Report [7], there were approximately 530,000 indigenous peoples represented by 16 distinctive tribes, accounting for 2.3% of the overall population. With regards to the geographical distribution, nearly 70% of the indigenous peoples do not dwell in metropolitan cities. The majority of Taiwanese indigenous peoples reside in rural townships or remote mountain areas. A large portion of older women inhabits these isolated, difficult-to-reach locations.

Previous national studies have shown that indigenous peoples in mountainous communities are socioeconomically disadvantaged, receiving and accessing little in terms of health care [8]. Compared with mainstream Taiwanese society, the indigenous group is highly vulnerable to multiple social welfare problems associated with poverty, substance abuse, alcoholism, low literacy, and low life expectancy [9-11]. Over the past 30 years, indigenous peoples had nearly 70% higher mortality than the general Taiwanese population [12]. Among remote indigenous peoples, malignant tumors caused the highest death rate, and the total number of indigenous cancer survivors has increased annually to approximately 15-20%, regardless of age and gender [7]. Survivors are those who have cancer diagnoses and are receiving or completed their cancer treatment.

In the past two decades, culture has been found to be an important factor influencing EOL planning [13], and ethnic differences during EOL treatment decision making have been widely documented [14-18]. Studies relevant to ACP and Taiwanese cancer survivors have been based almost entirely on nonindigenous populations and mainstream culture, such as the white racial majority in the United States.

According to recent studies published during national ACP campaigns in Taiwan, less than 5% of the entire Taiwanese population has been involved in ACP [19-22]. It is also unclear to what extent these findings, based on mainstream culture, could be generalized beyond the majority of Taiwanese people that are in the cities. Historically, individuals with limited health care access and low literacy have reported greater discomfort and less EOL participation than their counterparts in mainstream society [23-25]. Indigenous people’s traditional values may contradict basic values of patient autonomy and/or truth telling that underlie ACP, making their cultural needs distinct from the contemporary standard of EOL care [26-29].

In addition, searching for appropriate assessment of indigenous cancer patients’ ACP readiness is extremely challenging given the scarce literature [6,30]. Studies relevant to ACP of Taiwanese cancer survivors have been based almost entirely on non-indigenous population in mainstream culture, such as “the white racial majority” in the United States. Current national cancer studies in Taiwan have sampled less than 1% of the mountain indigenous cohort [7]; results are based on the majority of nonindigenous (Han tribe) plain residents. Reported percentages of cancer cohorts have been too small to use for examination of ACP readiness issues for all indigenous peoples.

In addition, studies related to indigenous peoples’ death and dying have been limited to epidemiological reports [9,31,32], funeral rituals, and/or local surveys [8,32]. Only one focus group attempted to address healthy indigenous teenagers’ cultural view of death [33], and one qualitative study explored Zhou tribes’ dilemmas of “a good death” [11]. However, neither included any ACP element. Only a few workshops have been held to introduce the principles of palliative care for this cultural group by the Hospice Foundation of Taiwan [34]. There has been little research to explore Taiwanese indigenous people’s intent to find public EOL education. It is likely that as a minority group, their tribal beliefs conflict with the values that underlie ACP [14,15,18,35-40], which ultimately influences their participation in public education.

As a result, the impact of cancer and tribal values on decisions regarding life-sustaining treatment and care remains understudied among Taiwanese pan-indigenous peoples. This cultural group’s intent and preference to participate in EOL and palliative care‒related interventions require extensive study, especially for those terminal cancer patients who will soon face difficult EOL decisions. Research specific to Taiwanese indigenous peoples is needed, in particular those who are geographically inaccessible, to understand how a terminal illness like cancer may change their EOL planning, and how their unique cultural beliefs, values, knowledge and attitudes about ACP differ, if at all, from indigenous peoples who have not had cancer.

Similarly, there has been little research to explore Taiwanese indigenous people’s intent to find public EOL education. It is likely that as a minority group their beliefs conflict with values associated with ACP [24,37,40-43]. Even though several studies were published after ACP became the subject of national campaigns [19-22], it is unknown to what extent these findings may be generalized beyond mainstream Taiwanese society.

The purpose of this study is to determine the possible impact of cancer as a life-threatening disease on Taiwanese indigenous peoples’ ACP readiness. Comparisons will be made between Taiwanese indigenous groups with and without existing cancer diagnoses; the hypothesis is that cancer survivors’ experiences before or during cancer treatment (or with no treatment) may differ. Issues specific to ACP can then be examined between groups similar in age and gender. In addition, this study will assess the impacts of obtained ACP knowledge, in relations to several conditions, including previous self-learning, earlier participation in any forms of ACP education, the registration of advance directives on the National Health Insurance Care, and prior care providing to dying family members.

This proposed study offers a unique opportunity to examine EOL-related decision making among mountain-residing indigenous cancer survivors who are geographically difficult to reach. For the first time, their current palliative care and hospice usage may be extensively surveyed. In addition, this study will be theoretically driven by the conceptual model of “Stage of Readiness for Change” [44,45] to explore facilitators and inhibitors affecting indigenous cancer survivors’ intentions. Specific intervention strategies may be discovered that are extremely helpful for developing a pioneering intervention that facilitates ACP public education participation for this cultural group.

## Methods

### Study Design

This descriptive study has a case-control design, in which a group of at least 100 Taiwanese indigenous cancer survivors will be compared with another 100 Taiwanese indigenous controls that have not had cancer. The survivor sample will consist of survivors of several common types of cancer, including malignant tumors and leukemia, and will be recruited from referral lists of oncological and family physicians of collaborative institutions from remote areas where indigenous populations reside. Controls will be selected from the same collaborative facilities in this study and will be matched to the survivor group in age and gender. Cancer diagnoses will be matched maximally so that comparisons may be made consistent across cancer types. Both cancer survivors and cancer-free controls will be surveyed for their willingness to participate in further public education in ACP. This also allows us to examine the effects of cancer as an exposure and also registry (and non-registry) of Do-Not-Resuscitate (DNR) status for these cancer types, which may have an influence on ACP readiness.

In the first year, the major research activities for this descriptive study will be gathering, preparing, and developing a valid and short questionnaire of readiness through in-depth interviews from a group of 10-20 indigenous cancer survivors. Interview guides will be developed in order to use phenomenological methods to describe indigenous cancer survivors’ EOL decision making, including their concerns, ACP knowledge, self-efficacy, and intent for ACP. Their preferred strategies whether to participate in a culturally appropriate intervention program will be explored.

In the second year, the main efforts will be in two dimensions: (1) case-control screening to reach an accrual goal of 100 subjects, in particular the identification of indigenous cancer survivors in remote, rural Taiwanese areas, and (2) partial data collection through face-to-face or telephone interviews to understand their ACP situations. Some data collection and analysis of the outcome variables (stages of readiness and intent) may be completed. Primary findings may be reported regarding associations among indigenous identities, demographic information, physical functioning, knowledge related to ACP, and an outcome of readiness according to the Theoretical Model.

Time and resources spent in the third year will be mostly on comparisons between factors such as knowledge related to ACP, their self-efficacy, and previous participation in ACP education, and the national registry status related to EOL planning. The complete results will be reported in the final year. The following section details sampling, recruitment, setting, data collection procedures, statistical considerations, and analytic methods.

### Sampling and Recruitment Setting

The potential cancer survivor pool was drawn through co-investigator physicians’ referral lists of participating sites and cancer registries of a few affiliated institutions where large Taiwanese indigenous populations reside and from areas where the most remote indigenous populations reside. Although this study was open to all large hospitals and affiliated institutions with substantial numbers of Taiwanese indigenous cancer survivors, it is expected that indigenous peoples are aggregated in certain Taiwanese rural areas. Four institutions and community branches of a teaching hospital in Northern, Western, and Eastern Taiwan have agreed to participate in the study and allowed access to cancer patients in the medical database.

The databases from which survivors were sampled were updated annually to include their current contact information. In the first year of the study, an effort was made based on the investigator’s judgment to recruit an interview sample varying in age, level of acculturation, socioeconomic status, and readiness for ACP. After discussing with two qualitative investigator experts, a minimum of 10 subjects (5 males and 5 females) was recommended for the in-depth interviews. However, because these 10 interviews might not provide sufficient saturation, the final qualitative sample size might be as large as 20 subjects (n=20). Saturation of data will be determined for each gender separately for this study. The inclusion criteria for interviewed cancer survivors were self-identified adult Taiwanese indigenous peoples above 20 years old, legally competent for advance care planning, fluent in Mandarin and/or Taiwanese, and currently treating or following up cancer at least 1 month prior to participation in this study. Excluded were those critically ill and legally incompetent for ACP during the data collection period. The investigator would contact potential subjects, and the study goal with the recruitment process will be explained in a room reserved for subjects’ convenience.

#### Cancer Survivors

Taiwanese indigenous cancer survivors who have been treated for various types of malignant tumors or leukemia will be recruited for participation in the second year of the survey study. The inclusion criteria for cancer survivors are self-identified Taiwanese indigenous peoples (n=100) residing in mountain areas (non-plains), currently treating or following up cancer at least 1 month prior to participation in this study. They must be legally competent for their own medical decisions without any predictable imminent medical crises. The cancer survivor sample will be drawn from physicians’ referral lists of affiliated institutions in areas of large Taiwanese indigenous populations.

Four institutions and community branches of the same teaching hospital mentioned above have agreed to participate in the study, provided ethical support, and allowed access to their cancer registry patients. If there are significant clusters of Taiwanese indigenous cases and survivors at other institutions, who meet the other criteria for study eligibility, we will add them and select controls from those areas proportionate to their representation in the survivor sample. However, based on the data received from these institutions, we are confident that this study can be completed at the four institutions that have agreed to participate.

Data recruitment will begin when the number of potential, eligible participants treated at each participant site is identified; survivors from the referral lists of each site will be conveniently selected later to create a matched case-control group within the study site. Names of survivors who are known to be at participating study sites will be drawn from database records. Efforts will be made in selection of cases to ensure, as much as possible, a balanced distribution across groups, for example, equal numbers or female and male for each cancer type. The four participating sites from which survivors will be sampled include updates of current contact information. The accuracy of the contacting information will be checked in this study, as well as to confirm that potential participants are still capable of participating in this study.

#### Controls

Several concerns must be considered in selecting controls. We chose the control sample from the same registry source as the cases to ensure both cancer cases and control survivors are representative of similar experiences, such as geographical areas, hospitals receiving treatment, and tribal identities. For this study, the cases will be drawn from several participating sites in geographically diverse areas in rural Taiwan. Since drawing controls from all other patient regions or the hospital census may be inefficient and costly, we have chosen to conveniently draw the controls from the same hospital base where the cancer survivors reside.

In order to avoid overmatching, the commonly used method of “frequency matching” on key variables will be used to ensure the equality of cancer survivors and controls among indigenous populations. The controls will be selected from the areas served by the participating institutions. They will be first oversampled to ensure a sufficient pool to allow frequency matching with cases/survivors, and later screening interviews will be conducted to identify eligible controls, that is, indigenous Taiwanese who have not had cancer. The inclusion criteria for the control group (n=100) are nonindigenous Taiwanese who are similar to the cancer survivors as a group in age and gender, but have not been diagnosed with any type of cancer. We will exclude participants who are not legally competent in executing ACP and are unable to be reached by the research team. During the screening interview, questions designed to classify controls by age and gender will be asked, and over the course of data collection, this information will be used to ensure the distribution of key variables so that the case/survivor group and control group may be sufficiently comparable.

### Instruments

Table 1 lists the instruments that will be developed and used to measure the variables for this study. Those valid instruments have been extensively used with minority ethnic populations. All of the instruments have been used successfully in telephone interviews, with the exception of one newly developed instrument (Stage of Readiness for Advance Care Planning), which has been used successfully in face-to-face interviews. The same instruments will be used for indigenous controls (who do not have cancer); however, the word “cancer” or any experiences relevant to cancer will be changed to “your health” wherever necessary. Medical characteristics, such as cancer stage at diagnosis, treatment received for cancer, and other related information will be obtained from medical files or cancer registry records at participating sites. Data regarding survivors’ and controls’ present health and ACP status will be obtained from the medical database as well.

**Table 1:** Constructs/components, variables, and measures.

Constructs/components	Variables	Quantitative measures
Readiness for ACP^a^ (outcome variable)	ACP Stage of change	Stage of change for ACP (based on an item algorithm)^a^
Physical functioning	General health status	Short Form-12 v2 Health Surveys Taiwan (Chinese) – Standard Recall (12 items) [46]
Quality of life	Life satisfaction	Life satisfaction subscale (33 items) of Quality of Life Index [47]
Demographics	Tribe, Age, Gender, Life-partnered, Income, Education, Employment, Preferred religion	Tribal identity, Age, Gender, Marital status, Income, Education, Work status, Religion
Knowledge obtained related to ACP	Knowledge about life-sustaining treatment and ACP; previous experiences related to EOL^c^	Knowledge of ACP scale (10 items)^a^; previous EOL experiences scale (6 items)^a^
Intent of Taiwanese indigenous peoples for ACP intervention	Preferred ACP program contents, settings and frequencies, learning methods, possible facilitators, and barriers.	Questions developed for soliciting subjects’ preferred strategies

^a^ACP: advance care planning.

^b^Quantitative measures included in the survey questionnaire were developed by Hsiung and Ferrans [28] based on the Transtheoretical Model of Health Behavioral Change [44] and previous ACP studies.

^c^EOL: end-of-life.

### Data Collection Procedures

The procedure used to collect data from both the cancer survivor cases and non-cancer controls are similar. The Principle Investigator will contact potential participants to describe the study and invite them to participate, and a telephone call will be scheduled to explain the study in detail and arrange a time for consecutive interviews. Interview schedules/questionnaires will be sent to their homes before the telephone interview, and both cases and controls are anticipated to complete the interviews by phone no more than two times. Telephone interviews with cases/survivors will continue only until the target sample size is reached.

### Statistical Considerations and Analytic Methods

#### Accrual Goals

A total of 200 Taiwanese indigenous peoples will be interviewed, including 100 indigenous cancer cases/survivors and 100 indigenous peoples who have not had cancer with key characteristics matched, similar to the survivor group. Required sample size depends on several parameters, such as desired power, alpha level, expected effect size, and a number of predictors. Normally, a power analysis is performed and effect size (f^2^) would be estimated to achieve this goal [48]. However, information needed for a power analysis and the effect size is not available from previous studies that have examined this phenomenon. A few instruments developed for this study have not been largely used across cultural groups. Therefore, Pedhazur and Schmelkin’s [49] recommendation of approximately 10 subjects per predictor for a reliable regression equation will be used in this pilot descriptive study. To understand the effect size that may then be used in a subsequent analysis for the categorical dependent variable with four levels, this pilot study would need a minimum number of at least 100 subjects of four groups each for the quantitative survey.

In addition, because there have been no such studies before, we could only provide a power estimate from the Quality of Life (QOL) Index. Previous studies have reported an average standard deviation of 4.5 for the QOL Index, with a difference of 2-3 points being a clinically meaningful difference. A difference of 2-3 points in the total score of the QOL Index has been associated with significant improvement in overall quality of life, self-image, physical symptoms, and general health in studies assessing change in quality of life. If the comparison in this study is based on 100 survivors and 100 control subjects, then there is approximately 80% to detect a 1-point difference in the QOL Index with a 2-tailed test conducted at the .015 level of significance. Our current size number for each group is greater than 99% power to detect a 2-point difference in the QOL Index. In comparing the survivor and control populations, the outcome of primary interest is Stage of Readiness for Advance Care Planning. In order to adjust for comparison, a type I error level of 0.05 will be used in the examination of power for this outcome.

#### Analytic Methods

To address selection biases, comparisons will be made between participants and those who fit the inclusion criteria yet decline to participate. In particular, we are interested in the differences in their demographic profiles and clinical characteristics. Basic patient descriptors will be described using *t* tests, Wilcoxon tests, and chi-square tests to make comparisons between eligible subjects who eventually do and do not participate in this study. Descriptive statistics stratified by survivorship status (subjects who are survivors or controls) will be used to describe the physical, psychological, social, economic profile of Taiwanese indigenous peoples, as well as the quality of life differences and the prevalence of readiness. Multiple regression models will be used to analyze the exposure effect of cancer, as an independent variable, on ACP readiness, as a dependent variable. While both are continuous measures, they will be analyzed using linear regression, unless a severe departure from normality is observed. If a highly skewed distribution is observed, an attempt will be made to analyze an appropriate transformation using linear regression, or ordinal categories will be defined and analyzed using the proportional odds logistic regression model. The coefficient for the effect of cancer will be tested against zero, adjusting for age and gender by including them in the model as additional independent variables.

In a second phase, potential mediating variables will be added to the model. Mediating variables will be considered in two blocks: (1) obtained knowledge related to ACP and (2) individual characteristics, including tribe identity. For each block, a model will first be fitted including all variables in that block. Second, a backward selection procedure will be used to remove insignificant variables according to the liberal criteria of *P*<.10, in order to capture potential changes in the effect of interest while still maintaining efficiency.

### Ethical Considerations

Before collecting data from subjects, permissions and ethical support to conduct this study have been obtained from the four affiliated institutions. The researcher has sought approval from the Institutional Review Board at Mackay Memorial Hospital, Taipei, in 2015. Careful attention will be paid to the sensitive nature of this study, and efforts will be made to ensure the protection of human subjects throughout the whole course. Upon recruitment, all potential participants will be given a written description of the purpose and data collection procedure of the study. This is to ensure that all potential participants understand the scope and purpose of this study. An opportunity will be provided during the informed consent process to allow clarification by participants. Although the research topic may be somewhat culturally sensitive, the probability and magnitude of harm or discomfort anticipated in this type of research is no greater than those risks ordinarily encountered in daily life. All documents related to participants’ responses are to be kept in a locked file cabinet accessible only to the researcher. All quantitative data will be kept for at least 5 years before being shredded or destroyed. When the results of this study are published or discussed in any conference and/or workshop, no information will be included that would reveal participants’ identities.

## Results

This study is expected to be completed within a 3-year timeframe, with results expected to be published in 2018.

## Discussion

### Principal Considerations

Since there has been no research on Taiwanese indigenous people’s aims for ACP, there is a need to understand the impact of survivorship on ACP readiness among those who are currently living with, through, and beyond cancer. This carefully designed study provides a unique opportunity because for the first time in Taiwan, cancer survivorship and ACP readiness for EOL planning will be examined among difficult-to-reach indigenous peoples. We expect to survey palliative care usage in remote indigenous groups, understand factors that influence ACP readiness, and foster culturally appropriate ACP public participation and policies in order to facilitate collaboration between cancer health care providers in various Taiwanese subcultures.

### Limitations

The greatest challenge of this cross-cultural case-control study is the identification, recruitment, and data collection of Taiwanese indigenous cancer survivors and controls in difficult-to-access areas. Connections have been made with physicians and cancer nurse case managers in rural Taiwanese areas where most indigenous peoples reside. Local assistants may be hired to foster case identification and control matches. In addition, while the influence of a stigma or fear related to death for cancer survivors and their comparatively healthy control cohorts is still unknown, the Principal Investigator will carefully deal with sensitive interview questions in order to explore death-related cultural beliefs and concerns. The use of small incentives has proved effective in the past to encourage participation and increase response in studies. The incentive value (<US $3) was determined after consulting a panel of indigenous leaders and cultural experts. As much as we want to believe that this incentive value may not be coercive enough as an inducement to create bias in the study, we are not entirely certain if providing any incentives would be an obvious limitation, considering poverty might be an issue among Taiwanese indigenous cancer survivors.

### Conclusion

This study will provide a better understanding of Taiwanese indigenous people’s cultural beliefs, current planning regarding their EOL medical and care preferences, and future projections of public ACP education participation in mainstream society. This will be of great use for health policy and resource allocation for Taiwan. Collaboration among affiliated hospitals in the Northern, Eastern, and rural areas will also facilitate consistent quality palliative care practice in Taiwan. All research personnel will be trained to obtain professional academic skills in data managing, data collection, and statistical analysis specifically for indigenous peoples. In addition, clinicians participating in this study will also obtain up-to-date information and resources about ACP, which is currently promoted at a national level in Taiwan.

## Appendix

Multimedia Appendix 1 Funding report from the Ministry of Science and Technology of Taiwan, Grant No: MOST 104-2511-S-715-002.

## Abbreviations

ACP: advance care planning
DNR: Do-Not-Resuscitate
EOL: end of life
QOL: quality of life
