# Supplementary material for: Exploring Advance Care Planning in Taiwanese Indigenous Cancer Survivors: Proposal for a Pilot Case-Control Study
Source: JMIR Res Protoc. 2017 Dec 21;6(12):e238. doi: 10.2196/resprot.5428 (PMC5754565; doi:10.2196/resprot.5428)
Supplement: Multimedia Appendix 1 [file resprot_v6i12e238_app1.pdf]

104年度 【 台灣原住民癌患參與「預立醫療自主計劃」公眾教育之準備度與意願初探：釐清文化信念、生活品質、先備知識與臨終醫療規劃相關性之病例對照研究 】經費核定清單

執行機構：馬偕學校財團法人馬偕醫學院
　　　　　財團法人臺灣基督長老教會馬偕紀念社會事業基金會馬偕紀念醫院

主　持　人：熊誼芳　　助理教授(護理學系)
共同主持人：白明忠　　主治醫師(內科)
　　　　　　方俊凱　　主治醫師(精神科)
　　　　　　李英芬　　講師(博士級、資深)(護理部)

補助項目	申請金額	核定金額	說　　明
業務費	349,000	450,000	一、研究人力費：200,000元<br>1. 碩士班研究生研究助學金1名60,000元<br>2. 臨時工資20,000元<br>3. 本部依規定主動增核研究主持費1名，月支10,000元(12.000月計)<br>二、耗材、物品、圖書及雜項費用：250,000元<br>三、本計畫彈性支用額度為11,500元
研究設備費	39,000	36,000	電腦及統計軟體
國外差旅費	90,000	60,000	一、出席國際學術會議：60,000元<br>二、本項目不核列管理費
管理費	31,040	29,000	研究主持費不核列管理費
合　　計	509,040	575,000	執行期限： 104/08/01 ～ 105/07/31<br>計畫編號： MOST 104-2511-S-715-002 -

研究類型：新進人員研究計畫(個別型)
研究性質：導向性基礎研究
應繳報告：期末報告
研究成果歸屬：馬偕學校財團法人馬偕醫學院
學門名稱：公民科技素養傳播與教育
流水號： 104WFDH350032
承辦人： 梅家瑜

各項費用之支用請依「科技部補助專題研究計畫經費處理原則」規定辦理。
